# Understanding patient journey in ulcerative colitis prior to biologic initiation: a 5-year exploration

**DOI:** 10.1186/s12876-021-01708-6

**Published:** 2021-03-17

**Authors:** Yiting Wang, Rupa Makadia, Christopher Knoll, Jill Hardin, Erica A. Voss, Daniel Fife, Kourtney Davis, Sheldon Sloan

**Affiliations:** 1 Janssen Research & Development, LLC, 1125 Trenton-Harbourton Road, Titusville, NJ 08560 USA; 2 Janssen Global Services, LLC, Raritan, 08869 NJ USA

**Keywords:** Ulcerative colitis, Corticosteroid exposure, Biologic initiation

## Abstract

**Background:**

There has been a more pronounced shift toward earlier, more aggressive therapies in Crohn’s disease than in ulcerative colitis (UC). The aim of this study was to describe the pre-biologic treatment and health care experience, including co-morbidities and overall health care utilization, for UC patients who initiated biologic therapies, in the 5 years prior to the initiation of the first biologic agent.

**Methods:**

UC patients who initiated a biologic agent approved for UC between 9/15/2005 and 1/30/2018 were identified from the IBM® MarketScan® Commercial Database, a large US database. The date of the first recorded UC biologic exposure was defined as the index date, and ≥ 5 years of pre-index records were required to evaluate patients’ treatment, disease progression and overall health care utilization prior to initiating biologic agents.

**Results:**

Among the 1891 eligible patients, treatment with oral corticosteroids, 5-aminosalicylates, and other non-biologic immunomodulators, all increased progressively across the 5 years prior to the index. From within year-five to within year-one prior to the index, the median duration of oral corticosteroid treatment increased from 34 to 88 days per year and the proportion of patients who experienced more extensive/pancolitis disease increased from 16 to 59%. Overall, the frequency of all-cause health care visits also increased.

**Conclusions:**

Patients with UC experienced increasing morbidity and treatment burden in the 5 years prior to initiating biologic therapy. To achieve reduced corticosteroids in UC management, better risk stratification is needed to help identify patients for more timely biologic treatment.

**Supplementary Information:**

The online version contains supplementary material available at 10.1186/s12876-021-01708-6.

## Introduction

Crohn’s disease (CD) and ulcerative colitis (UC) are the two major forms of inflammatory bowel disease (IBD). CD is considered in most cases as a chronic, progressive, destructive disease [1]. CD therapies were previously limited to conventional immunomodulators (e.g., thiopurines) [2] until the late 1990s, when the introduction of anti-tumor necrosis factor (TNF) biologic agents created a paradigm shift in CD management [3, 4], a shift toward earlier, more aggressive treatment with the aim of achieving “deep remission”, inducing “mucosal healing”, thus preventing or retarding progression to irreversible bowel damage[5].

UC is characterized by diffuse mucosal inflammation limited to the colon. UC treatment guidelines set a goal of induction and maintenance of remission of symptoms to provide an improved quality of life, reduction in need for long-term corticosteroids, and minimization of cancer risk (long-term mucosal healing may reduce the risk of dysplasia) [6]. Historically, 5-aminosalicylates (5-ASA) have been used as first-line therapy in patients with moderately to severely active UC with or without the addition of corticosteroids. For patients who have failed to respond to these oral medications and hospitalized for intensive treatment, colectomy may have to be considered if patients do not respond to intravenous corticosteroids by day 3, 7, or 21 of treatment (clinical decision weighing the benefits and risk in the context of “save the patient” or “save the colon”) [7]. More recently, however, for adults with moderate to severe ulcerative colitis in the outpatient setting, the American Gastroenterological Association (AGA) suggests early use of biologic agents with or without immunomodulator therapy, rather than gradual step-up therapy after failure of 5-ASA [8]. In hospitalized adult patients with acute severe UC, the AGA suggests using intravenous methylprednisolone dose equivalent of 40 to 60 mg/d [8]. Despite the availability of potent biologic agents to induce and maintain remission for UC patients, and even as rescue therapy (i.e., last resort before colectomy) [9], it has been claimed that surgery is still required for 20%-30% of patients with UC at some point during their lifetime [10]. Population-based inception cohort studies have reported 5-year cumulative risk of UC disease progression of about 13%, and significantly higher risk of colectomy in patients with extensive colitis [11].

In contrast to CD, there is a general inertia to initiate biologic agents early for UC treatment. Biologic use overall is about 44% in CD compared to 16% in UC among commercially insured patients in the US [12]. While this possibly reflects difference in disease progression between CD and UC at least for some patients, it is also possibly related to the lack of real-world evidence in support of early biologic treatment to lower long-term risk of colectomy in UC, despite evidence from clinical trials for reduced hospitalization and surgery with infliximab [13–15]. On the other hand, the common practice of delayed biologic treatment in UC would present challenges such as confounding by disease severity to real-world evidence generation. Further, there is a lack of reliable and practical clinical measures to assess the progression of bowel damage in UC and the impact of disease-modifying agents in preventing it [16]. Therefore, some patients with UC may not receive biologics until there is severe morbidity and failure of prolonged courses of corticosteroids and immunomodulators.

The present study aims to describe the treatment and health care utilization patterns of UC patients who initiated biologic therapies in the 5 years prior to the initiation of the first biologic agent, and to understand whether these patients had evidence of a refractory disease course of chronic recurrent flares in routine clinical practice before they were initiated on biologic therapies.

## Methods

### Data source

A retrospective, descriptive, observational cohort study was conducted using data from the IBM MarketScan® Commercial Database (CCAE). The CCAE is a US database that includes adjudicated health insurance claims (e.g., inpatient, outpatient, and outpatient pharmacy) as well as enrollment data from large employers and health plans who provide private healthcare coverage to employees, their spouses, and dependents. We chose this database for its large size and comprehensive data capture of healthcare encounters and services. The number of people covered in CCAE represents 3.2% and 3.4% of the US citizens with employment-based health insurance coverage in 2016 and 2017, respectively [17]. The CCAE database has been converted to the Observational Medical Outcomes Partnership (OMOP) Common Data Model (CDM) [18] version 5.3.1, which includes a standard representation of health care experiences (such as information related to drug utilization and condition occurrence), as well as common vocabularies for coding clinical concepts and enables consistent application of analyses across multiple disparate data sources [19].

### Study sample

Patients with UC who initiated a biologic agent approved for UC (infliximab, adalimumab, golimumab, vedolizumab) between 9/15/2005 (approval date of infliximab for UC indication) and 10/31/2018 (last date of available data) were identified. For each biologic initiator, the date of the earliest pharmacy or procedure record for a UC biologic was defined as the index date for that patient. The biologic initiators were required to be bio-naïve prior to the exposure index date, i.e., with no exposure to any UC biologics or biologics indicated for other related autoimmune conditions (rheumatoid arthritis, psoriasis) from all available health care claims. The definition for UC required two or more physician encounters with a UC diagnosis recorded on different dates prior to or on the exposure index date [20]; or one or more recorded UC diagnoses plus one or more prescription dispensings for azathioprine, 6-mercaptopurine, or aminosalicylates (i.e., balsalazide, mesalamine, olsalazine, sulfasalazine), within the 1 year prior to the index date [21] (see Additional file 1: Fig. S1 for an evaluation of the empirical accuracy of this algorithm, including the diagnosis codes for CD and UC).

To reduce misclassification between UC and Crohn’s disease, for patients having both CD and UC diagnoses recorded prior to or on the index date, we only included patients for whom the most recent record was a UC diagnosis and the number of UC diagnoses exceeded the number of CD diagnoses within the 1 year prior to the index date [22–24]. In a sensitivity analysis, we evaluated the frequency and recency of diagnosis records for UC and CD within 1 year both before and after the index date (see Additional file 1: Fig. S1).

The analytical sample included adult patients (i.e., age 18 years or older on the index date) who had at least 5 years of pre-index enrollment history in the database, with the earliest record for UC diagnosis and/or UC treatment at least 5 years prior to the index date. To reduce the likelihood of including patients who initiated the biologics mainly for non-UC indications, patients with a physician diagnosis of rheumatoid arthritis, ankylosing spondylitis, psoriasis or psoriatic arthritis within 1 year prior to or on the index date were excluded. To focus on evaluation of UC disease burden, we also excluded patients with two or more diagnosis records of malignancy (except for non-melanoma skin cancer) at any time prior to the index date. The requirement of two or more records was intended to avoid excluding patients who had only one diagnosis code that represented cancer screening or diagnosis rule-out rather than true malignancy.

### Analysis

As illustrated in Additional file 1: Fig. S1, UC patient treatment and health care experience were described over the entire 5 years prior to the index date, as well as for each of the 5 years individually.

UC patient treatment included oral corticosteroids, 5-ASA, and other non-biologic immunomodulators (IMM), as listed in Additional file 1: Table S1. During each of the time periods specified above, UC treatment exposure was summarized as yes or no according to the presence or absence of a prescription record. For patients with an exposure to a UC treatment, cumulative duration of exposure to that treatment was evaluated by summing up days-supplied from every prescription record for oral formulations, or as 15 days per intravenous injection [25]. If days-supplied was missing from some but not all pharmacy claims for a patient, it was imputed as the mode of (non-missing) days-supplied from corticosteroid prescriptions for the patient; if missing from all pharmacy claims for a patient, it was imputed as the mode of (non-missing) days-supplied from corticosteroid prescriptions for all other patients. Total cumulative dose of oral corticosteroids was estimated as total prednisone-equivalent milligrams, a dose conversion table for different oral corticosteroids is provided in Additional file 1: Table S2. Imputation for missing data on dose was done in the same way as for missing days-supplied data. The overall percentage of zero values of corticosteroid days-supplied or milligrams prescribed was low (< 5% missing for each, and < 1% missing both), so no correction or imputation was done for zero values of days-supplied or dose. We also evaluated the number of different 5-ASA products prescribed for the UC patients to understand whether there existed any cycling in the use of these drugs. All-cause health care utilizations were summarized by the numbers of outpatient, inpatient, and emergency department visits.

We also estimated the pre-index, cumulative prevalence of comorbidities among the UC patients. All diagnosis records (including more than 5 years prior to index date, if available) for anemia, diabetes, hypertension and stroke were searched up to the last day of each of the five pre-index periods (see Additional file 1: Fig. S1). The distribution of UC disease extent among the patients was evaluated during each of the pre-index 5-year period by the following categories, (1) extensive/pancolitis, (2) left-sided, (3) proctitis/proctosigmoiditis, or (4) missing/unknown, based on UC diagnosis codes in a hierarchical order. For example, if a patient’s most extensive disease code in year-5 was for left-sided colitis based on all available UC diagnosis records in the past, left-sided colitis was designated as the category of disease extent, and would be carried forward to year-4, year-3, and so on, until a diagnosis code for extensive/pancolitis was found, from which point on the patient’s UC disease extent would then be categorized as extensive/pancolitis. If no codes were available for any of the specific UC disease extent categories, patients were grouped to the missing/unknown category. We also evaluated the distribution of UC disease extent among patients who had at least one diagnosis code for disease extent during each of the 5 years pre-index.

For continuous variables, mean, standard deviation, median, the 25^th^ and 75^th^ percentile were provided; for categorical variables, percentages were provided. We did not pre-specify specific hypothesis for statistical testing.

### Ethical considerations

The use of the IBM® Marketscan® databases was reviewed by the New England Institution Review Board (IRB) and was determined to be exempt from broad IRB approval. Informed consent is not applicable as this research project did not involve human subject research.

## Results

### Study patients

A total of 1891 eligible patients were identified, with mean (median) age of 47 (48) years at biologic initiation (range 18–65, interquartile range 38–56 years), and 49% (n = 934) were male. A study sample creation flowchart is provided in Additional file 1: Fig. S2.

### Pre-index UC medications

Across the 5 years prior to starting a biologic, treatment with oral corticosteroids, 5-ASA, and other non-biologic IMM increased progressively. The median annual duration of oral corticosteroids increased from 34 to 88 days from year-5 to year-1 (i.e., within 1 year) prior to biologic initiation, with the biggest increase observed from year-2 to year-1 (from a median of 52 to 88 days). A similar pattern was observed for prednisone-equivalent milligrams of corticosteroid exposure, which had the biggest increase from year-2 to year-1. During the 5 years prior to biologic initiation, the UC patients had a mean (median) exposure of 2972 (1758) milligrams of prednisone (equivalent). The proportion of patients receiving 5-ASA prescriptions increased from 59 to 85% from year-5 to year-1, with a 19% increase from year-2 to year-1 alone. We did not observe a pattern of cycling through different 5-ASA products, with an average of 2.8 different products used out of 22 available products and formulations (see Additional file 1: Table S1); still, the duration of 5-ASA treatment was substantial, with a mean (median) exposure of 856.8 (796) days. The proportions of patients receiving non-biologic immunomodulators other than 5-ASA increased from 18 to 37% from year-5 to year-1, with a 14% increase from year-2 to year-1. Over the 5 years, almost half (46%) of the patients were also prescribed non-biologic immunomodulators other than 5-ASA, with a mean (median) exposure of 564.4 (300) days. There was a decrease in the mean (median) duration of treatment with 5-ASA and other non-biologic immunomodulators from year-5 to year-4, parallel to an increase in both the proportion of patients taking these medications, and the mean (median) duration of corticosteroids (Table 1).

**Table 1:** Pre-biologic health care experience among patients with ulcerative colitis, n = 1891

	Time window prior to biologic exposure index date^a^ (calendar date range, pre-index days covered)					
	Year-5 (− 1825 to − 1461)	Year-4 (− 1460 to − 1081)	Year-3 (− 1080 to − 721)	Year-2 (− 720 to − 366)	Year-1 (− 365 to − 1)	All 5 years (− 1825 to − 1)
Oral corticosteroids exposure						
≥ 1 prescription, n (% of 1891)	603 (32)	663 (35)	752 (40)	874 (46)	1571 (83)	1781 (94)
Duration of exposure, days						
Mean (SD)	70.8 (87.9)	69.0 (86.1)	70.4 (89.7)	79.5 (86.6)	108.4 (88.9)	214.0 (257.9)
Median (IQR)	34 (12–90)	34 (12–90)	36 (12–90)	52 (21–106)	88 (49–146)	145 (71–254)
Prednisone equivalent milligrams						
Mean (SD)	743.8 (987.4)	824.5 (1343.2)	932.7 (1608.1)	1132.9 (1733.8)	1659.2 (1906.7)	2972.0 (4160.5)
Median (IQR)	360 (120–920)	360 (114–950)	384 (120–1050)	570 (190–1298)	1080 (510–2130)	1758 (824–3540)
5-Aminosalisylates (5-ASA)						
≥ 1 prescription, n (% of 1891)	1119 (59)	1176 (62)	1211 (64)	1304 (69)	1608 (85)	1768 (93)
# of different products/formulations						
Mean (SD)	1.5 (0.7)	1.5 (0.7)	1.5 (0.8)	1.5 (0.7)	1.8 (0.9)	2.8 (1.4)
Median (IQR)	1 (1–2)	1 (1–2)	1 (1–2)	1 (1–2)	2 (1–2)	3 (2–4)
≥ 1 prescription of oral 5-ASA, n (% of 1891)	1077 (57)	1135 (60)	1162 (61)	1244 (66)	1557 (82)	1731 (92)
Duration of oral 5-ASA, days						
Mean (SD)	246.1 (117.2)	237.4 (118.6)	245.1 (121.3)	243.2 (119.7)	232.1 (123.7)	856.8 (613.6)
Median (IQR)	270 (150–345)	270 (150–330)	270 (150–360)	270 (150–360)	240 (120–330)	796 (270–1380)
Other immunomodulators (IMM)^b^						
≥ 1 oral prescription, n (% of 1891)	343 (18)	357 (19)	376 (20)	440 (23)	700 (37)	879 (46)
Duration of other oral IMM, days						
Mean (SD)	234.5 (125.9)	239.3 (123.1)	238.7 (125.4)	231.1 (125.8)	198.2 (132.3)	564.4 (570.3)
Median (IQR)	270 (120–360)	270 (150–360)	270 (120–360)	252 (112–360)	180 (70–324)	300 (90–960)
All-Cause Health care utilization						
≥ 1 outpatient visit, n (% of 1891)	1833 (97)	1834 (97)	1849 (98)	1845 (98)	1889 (100)	1891 (100)
Outpatient visits, # of times						
Mean (SD)	14.4 (14.3)	14.8 (13.8)	15.8 (16.2)	17.0 (15.8)	22.8 (15.4)	83.0 (59.8)
Median (IQR)	10 (5–18)	11 (6–19)	12 (6–20)	12 (7–22)	19 (2–29)	4 (1–103)
≥ 1 Emergency department visits, n (% of 1891)	354 (19)	401 (21)	381 (20)	416 (22)	692 (37)	1241 (66)
Emergency department visit, # of times						
Total	554	668	594	667	1295	3778
Mean	1.6	1.7	1.6	1.6	1.9	3.0
≥ 1 inpatient stay, n (% of 1891)	153 (8)	152 (8)	168 (9)	183 (10)	475 (25)	824 (44)
In-patient stays, # of times						
Total	208	201	239	238	672	1558
Mean	1.4	1.3	1.4	1.3	1.4	1.9
≥ 1 inpatient stay with an infection, n (% of 1891)	20 (1)	33 (2)	29 (2)	48 (3)	125 (7)	230 (12)
Hospitalized infections, # of times						
Total	31	60	74	115	234	514
Mean	1.6	1.8	2.6	2.4	1.9	2.2

SD, standard deviation; IQR, interquartile range

^a^Year-5 pre-index included all days between (index date − 5 × 365 days) and (index date − 4 × 365 − 1 days); year-4 pre-index included all days between (index date − 4 × 365 days) and (index date − 3 × 365 − 1), and so on, and year-1 pre-index included all days between (index date − 365 days) and (index date -1)

^b^Other immunomodulators included thiopurines, methotrexate and cyclosporine, tacrolimus, thalidomide and mycophenolate mofetil

### Pre-index all-cause health care utilizations

All-cause health care visits across outpatient, emergency department and inpatient settings all increased among the UC patients across the 5 years prior to biologic initiation. All patients (100%) had an outpatient visit (for any reason), but the number of outpatient visits was skewed, with mean (SD) of 83 (59.8) visits across the 5 years, and median (interquartile range (IQR)) 4 (1–103) visits. Across the 5 years, 66% of the patients had at least one emergency department visit, 44% of patients had at least one inpatient stay, and 12% were hospitalized with an infection diagnosis (Table 1).

### Pre-index comorbidities

Across the 5 years, the cumulative prevalence of the comorbidities all increased from year-5 to year-1, from 9.6 to 41.5% for anemia, from 5.7 to 13.6% for diabetes, from 4.7 to 22.3% for hypertension, from 0.2 to 2.6% for stroke, and from 1.3 to 6.8% for osteoporosis (Table 2).

**Table 2:** Cumulative prevalence of comorbidities and ulcerative colitis disease extent

	Checking all available history up to (the last day of) each period				
	Year-5 (-1461)	Year-4 (-1081)	Year-3 (-721)	Year-2 (-366)	Year-1 (-1)
Comorbidities, n (% of 1891)					
Anemia	182 (9.6%)	288 (15.2%)	397 (21.0%)	517 (27.3%)	784 (41.5%)
Diabetes	107 (5.7%)	136 (7.2%)	173 (9.1%)	213 (11.3%)	257 (13.6%)
Hypertension	88 (4.7%)	177 (9.4%)	258 (13.6%)	335 (17.7%)	422 (22.3%)
Stroke	4 (0.2%)	12 (0.6%)	22 (1.2%)	36 (1.9%)	50 (2.6%)
Osteoporosis	25 (1.3%)	57 (3.0%)	77 (4.1%)	102 (5.4%)	129 (6.8%)
UC extent (hierarchical)^a^, overall^b^					
5-year UC diagnosis record, N (%)	1885 (100)	1885 (100)	1885 (100)	1885 (100)	1885 (100)
Extensive/pancolitis, n	298 (16)	450 (24)	562 (30)	720 (38)	1117 (59)
Left-sided, n	112 (6)	168 (9)	196 (10)	215 (11)	272 (14)
Proctitis/Proctosigmoiditis, n	88 (5)	127 (7)	156 (8)	179 (9)	164 (9)
Not available, n	1387 (74)	1140 (60)	971 (52)	771 (41)	332 (18)
UC extent (hierarchical)^a^, subset^c^					
5-year UC diagnosis record, N (%)	498 (100)	498 (100)	498 (100)	498 (100)	498 (100)
Extensive/pancolitis, n	298 (60)	317 (63)	337 (68)	357 (78)	388 (78)
Left-sided, n	112 (22)	106 (21)	101 (20)	89 (18)	75 (15)
Proctitis/Proctosigmoiditis, n	88 (18)	75 (15)	60 (12)	52 (10)	35 (7)

^a^UC disease extent assigned hierarchically based on diagnosis codes, i.e., precedence given to codes for more extensive disease and carried forward in time

^b^Among the total of 1891 patients, six did not have a record of UC diagnosis within the five years prior to initial biologic with post-UC diagnosis status inferred by records of UC treatment alone

^c^Among the total of 1885 patients above, 498 had at least one diagnosis record for UC disease extent within each of the 5 years prior to initial biologic

### Pre-index UC disease extent

The proportion of patients who experienced more extensive/pancolitis UC disease increased from 16% in year-5 to 59% in year-1 prior to biologic initiation. Among patients with non-missing disease extent during each of the 5 years pre-index, the proportion of patients who experienced more extensive/pancolitis UC disease increased from 60% in year-5 to 78% in year-1 prior to biologic initiation (Table 2).

## Discussion

In this large US commercial insurance claims database, we observed that patients with UC experienced significant morbidity and health care burden in the 5 years prior to the initiation of a biologic for UC treatment, as exemplified by the progressive increase in the use of non-biologic medications (i.e., duration and dose of corticosteroids, 5-ASA and other immunomodulators), health care resources (i.e., the number of all-cause emergency department, inpatient and outpatient visits), and overall worsening UC disease extent as well as more prevalent comorbidities. Although the data represented are surrogates for active disease/flares in the five years prior to initiating biologics, they suggest a possible health and health care burden penalty for delaying biologic therapy.

Our study findings support the latest guideline of early use of biologic agents rather than gradual step-up after failing conventional therapies for adult patients with moderate to severe UC [8]. It should also be noted that UC disease and comorbidities all progressed considerably over the 5 years even for the patients who eventually initiated biologics. For example, anemia was prevalent in 41.5% of the patients (specifically, 39.4% of men and 55.3% of women) within 1 year prior to biologic initiation. This was more than 6 times higher than the overall anemia prevalence of 5.6% in the general US population [26]. This may reflect poorly controlled disease and poor nutrition as a result of abdominal symptoms and possibly blood loss due to rectal bleeding. The prevalence of diabetes increased from 5.7 to 13.6% during the 5 years that led up to the biologic initiation, which exceeded the overall diabetes prevalence in the general US population [27]. The prevalence of hypertension also increased from 4.7 to 22.3% during the 5 years prior to biologic initiation. The prevalence of osteoporosis increased (from 1.3%) to 6.8% at biologic initiation among the UC patients in the present study, while prevalence of less than 3% would be expected in general US population with private insurance coverage with the same age and sex distribution [28]. The mechanisms for the higher observed prevalence of comorbidities in UC patients are beyond the scope of this study but may be associated with corticosteroids’ side effects. For example, a study of six inflammatory diseases (including 25,162 patients with inflammatory bowel disease) found that cumulative dose of oral glucocorticoids was associated with increased incidence of hypertension [29]; another study of patients with rheumatoid arthritis found that daily dose of ≥ 7.5 mg of glucocorticoids was associated with a hazard ratio of 2.33 (95% CI 1.68–3.22) for incident diabetes compared with patients not prescribed oral glucocorticoids [30]. Osteoporosis is also an important consideration for patients with increased dose and duration of corticosteroids exposure [31, 32]. Our present study only described a positive trend of increasing comorbidity prevalence that correlated with increasing cumulative dose of corticosteroids and other immunosuppressants, further research is needed to evaluate the specific associations between cumulative exposure to corticosteroids and the incidence of diabetes, hypertension, and osteoporosis. Confounding by poorly controlled UC disease should also be considered in such association studies.

The strengths of the study include the use of a large insurance claims database and nearly two thousand UC patients with at least 5 years of pre-biologic health care administrative history. We did not find previous published studies that evaluated the patient journey in terms of UC and overall health care experience for such a long period. Our analysis revealed a progressively worsening disease pattern among UC patients before the initiation of biologics, which is consistent with the relatively low rate of biologic use in UC [12], and may in part help explain why observational studies to date did not find reduced colectomy risk in UC with “early” biologic treatment, despite evidence from clinical trials for reduced hospitalization and surgery with infliximab [14–16].

There are also important limitations to consider for this study. The CCAE claims database does not capture in-hospital medication prescriptions, therefore we ignored inpatient exposure to corticosteroids and other medications. We also did not impute days supplied for patients whose claims record had a zero value. These could all result in underestimation of UC treatment exposure.

Another limitation to consider is the generalizability of the study. First, the CCAE commercial claims database only includes people with employment-based, private healthcare coverage, and our study population may not be representative of patients covered by Medicaid and Medicare. In patients with IBD overall, insurance coverage for biologic agents in commercial plans may be more extensive than other plans. Second, the median (average) duration of enrollment of all patients with a UC diagnosis during the study period is about 3.7 (4.8) years in the CCAE database, and our study sample of patients which required at least 5 years of pre-biologic UC history (see Additional file 1: Fig. S2) may not be representative of patients with shorter enrollment. Five years of pre-biologic data still were inadequate for the determination of when the patients had initial diagnosis of UC or time to initial biologic treatment since UC diagnosis..

An additional limitation by design is that the present study describes only the experience of patients who received biologic agents. The experience of other patients who did not receive such agents was likely quite different and the clinical usefulness of the results of the present study is limited by the fact that clinicians may not be able to reliably predict which patients will progress to require biologic agents.

Key challenges with treating UC patients with biologics early include both costs and uncertainty about the disease course, i.e., some patients may remain in long-term remission whereas others progress. An emerging goal in UC management is that of mucosal healing. To achieve that in addition to reducing the need for long-term corticosteroids, understanding of the most effective diagnostic, treatment, and preventive strategies is necessary [33]. Therefore future research should evaluate patient experience prior to colectomy to understand if most or some patients with UC were initiating biologic agents as a ‘rescue’ therapy or last resort prior to the surgery, and build patient-level predictions to help provide a clinically useful predictor for UC disease progression. Furthermore, it will be useful to study whether important clinical outcomes such as corticosteroid-free remission, colectomy and hospitalization rates, as well as serious adverse events, vary by cumulative exposure to corticosteroids as well as other non-biologic therapies. These are important real-world evidence gaps that can inform treatment decisions by patients and clinicians [34–36].

## Conclusions

Our study in a large US commercially insured population found substantial disease burden and progression among UC patients in the 5 years prior to initiation of biologic agents. Cumulative exposure to corticosteroids may not have been widely recognized, and better disease control is warranted. To achieve treatment guideline-recommended reduced steroid in UC management, better risk stratification is needed to help identify patients who can benefit from timely biologic treatment.

## Supplementary Information

**Additional file 1.** Details of UC patient journey analysis.

## Data Availability

The data underlying this article were made available to the authors by third-party license from IBM MarketScan®, a commercial data provider in the US. Under the licensing agreement, the authors cannot provide the raw data themselves. Other researchers could access the data by purchase through IBM MarketScan®; and the inclusion criteria specified in the Methods section would allow them to identify the same cohort of patients we used for these analyses. Interested individuals may see https://www.ibm.com/products/marketscan-research-databases/databases for more information on accessing the MarketScan data. We confirm that no authors had special privileges to access data from IBM MarketScan via third-party license, and that other researchers would be able to access the data in the same manner as the authors.

## Abbreviations

UC: Ulcerative colitis
CD: Crohn’s disease
IBD: Inflammatory bowel disease
TNF: Tumor necrosis factor
5-ASA: 5-Aminosalicylates
AGA: American Gastroenterological Association
CCAE: IBM MarketScan® Commercial Database
OMOP: Observational Medical Outcomes Partnership
CDM: Common Data Model
IMM: Immunomodulators
IQR: Interquartile range
