# Supplementary material for: Understanding patient journey in ulcerative colitis prior to biologic initiation: a 5-year exploration
Source: BMC Gastroenterol. 2021 Mar 17;21:121. doi: 10.1186/s12876-021-01708-6 (PMC7967955; doi:10.1186/s12876-021-01708-6)
Supplement: Supplementary file 1 — Additional file 1. Details of UC patient journey analysis. [file 12876_2021_1708_MOESM1_ESM.docx]

Title page for supplementary file

**Manuscript complete title:**  Understanding Patient Journey in Ulcerative Colitis Prior to Biologic Initiation: A 5-Year Exploration

**Manuscript short title:** Ulcerative Colitis Patient Journey

**Authors:**

Yiting Wang, Janssen Research & Development, LLC, Email: [ywang28@its.jnj.com](mailto:ywang28@its.jnj.com)

Rupa Makadia, Janssen Research & Development, LLC, Email: [rmakadia@its.jnj.com](mailto:rmakadia@its.jnj.com)

Christopher Knoll, Janssen Research & Development, LLC, Email: [cknoll1@its.jnj.com](mailto:cknoll1@its.jnj.com)

Jill Hardin, Janssen Research & Development, LLC, Email: [jhardi10@ITS.JNJ.com](mailto:jhardi10@ITS.JNJ.com)

Erica A. Voss, Janssen Research & Development, LLC, Email: [EVoss3@its.jnj.com](mailto:EVoss3@its.jnj.com)

Daniel Fife, Janssen Research & Development, LLC, Email: [DFife@its.jnj.com](mailto:DFife@its.jnj.com)

Kourtney Davis, Janssen Research & Development, LLC, Email: [KDavis24@its.jnj.com](mailto:KDavis24@its.jnj.com)

Sheldon Sloan, Janssen Global Services, LLC, Email: [sheldonsloan1@gmail.com](mailto:sheldonsloan1@gmail.com)

**Address for correspondence:** Yiting Wang, 1125 Trenton-Harbourton Road, Titusville, NJ 08560, USA, Telephone: 1-302-740-5195.

# **Supplementary Figures and Tables**

## **Supplementary Figure 1. Empirical evaluation of the accuracy for ulcerative colitis (UC) identification algorithm**

For the empirical evaluation of the accuracy for UC definition from the claims database, we included all patients (n=10,969) who were classified as having UC on the index date, and did not restrict to the smaller subset of patients who had at least five years of post-UC enrollment history.

From the n=10,969 patients, n=7,054 (64.3% of 10,969) had at least 1 year of enrollment data after index date, with an estimated post-index classification accuracy was ((4313+6120) + (1125+422))/7054=92% overall.


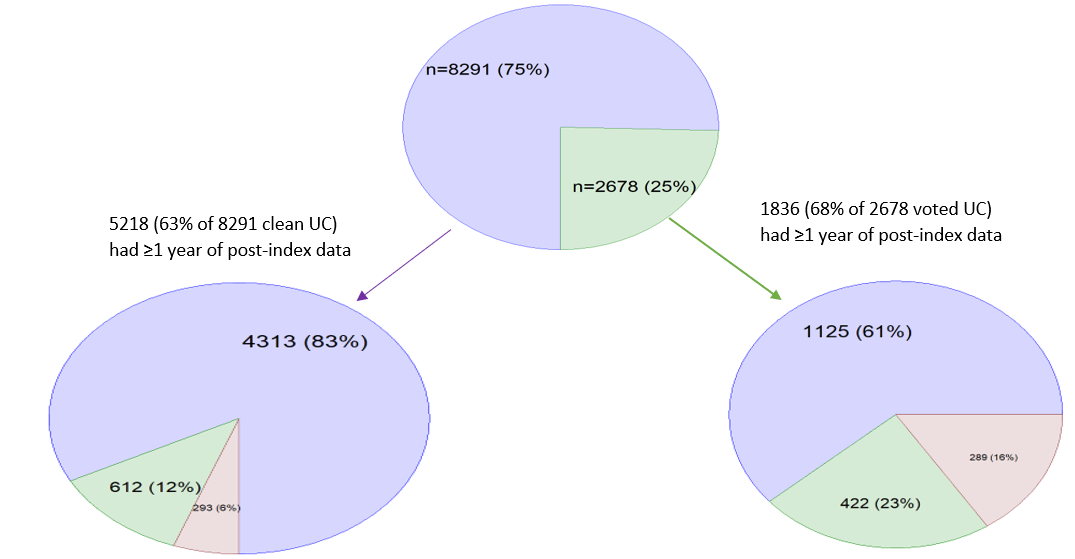


Color legend: Purple, no Crohn’s diagnosis ever before or after index; Green, Crohn’s diagnosis before or after index but still voted as UC by majority of UC coding frequency and recency criteria; Maroon, post-index Crohn’s diagnosis and not voted as UC by both the recency and majority criteria.

International Classification of Disease (ICD) diagnosis codes:

- CD, ICD-9-CM, 555; ICD-10-CM, K50
- UC, ICD-9-CM, 556 (556.6 for extensive/pancolitis; 556.5 for left-sided; 556.2 and 556.3 for proctitis/proctosigmoiditis); ICD-10-CM, K51 (K51.0 for extensive/pancolitis; K51.5 for left-sided; K51.2 and K51.3 for proctitis/proctosigmoiditis)

## **Supplementary Figure 2. Cohort creation flow chart**


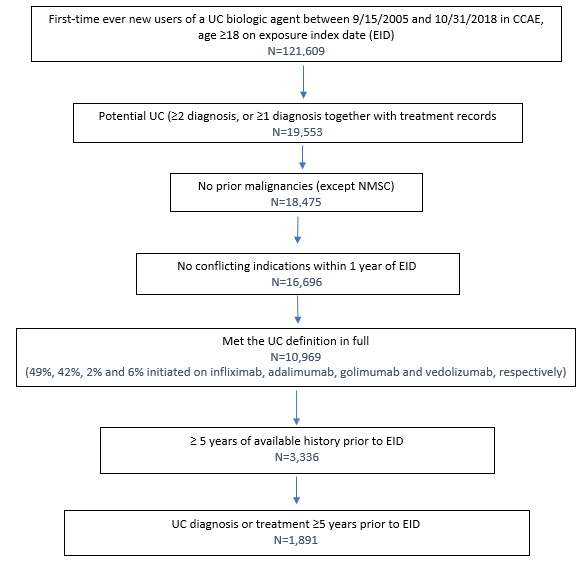


## **Supplementary Table 1. 5-ASA products and formulations, 22 combinations**

| **Compound (molecular formula)** | **Product name** | **Formulations** |
| --- | --- | --- |
| Balsalazide (C_17_H_15_N_3_O_6_) | Balsalazide disodium | capsule |
|  | Balsalazide disodium | tablet |
|  | Colazal | capsule |
|  | Giazo | tablet |
| Mesalamine (C_7_H_7_NO_3_) | Apriso | Capsule |
|  | Asacol HD | Tablet |
|  | Canasa | Suppository |
|  | Delzicol | Capsule |
|  | Lialda | Tablet |
|  | Mesalamine | Enema |
|  | Mesalamine | Tablet |
|  | Mesalamine | Suppository |
|  | Mesalamine | Capsule |
|  | Pentasa | Capsule |
|  | Rowasa | Enema |
|  | Rowasa | Suppository |
|  | Sfrowasa | Enema |
| Olsalazine (C_14_H_10_N_2_O_6_) | Dipentum | Capsule |
| Sulfasalazine (C_18_H_14_N_4_O_5_S) | Azulfidine | Tablet |
|  | Azulfidine EN-tabs | Tablet |
|  | Azulfidine EN-tabs | Tablet, delayed release |
|  | Sulfasalazine | Tablet |

## **Supplementary Table 2. Corticosteroids Conversion**

| **Compound** | **Anti-inflammatory /immunosuppressive potency** | **Prednisone-equivalent dose** | **Multiplication factor to derive prednisone-equivalent mg** |
| --- | --- | --- | --- |
| Betamethasone | 25.00 | 0.80 | x 6.25 |
| Budesonide | 16.00 | 1.25 | x 4.00 |
| Cortisol/Hydrocortisone | 1.00 | 20.00 | x 0.25 |
| Cortisone | 0.80 | 25.00 | x 0.20 |
| Deflazacort | 2.70 | 7.50 | x 0.67 |
| Dexamethasone | 25.00 | 0.80 | x 6.25 |
| (6alpha-) methylprednisolone | 5.00 | 4.00 | x 1.25 |
| Paramethasone | 10.00 | 2.00 | x 2.50 |
| Prednisolone | 4.00 | 5.00 | x 1.00 |
| Prednisone | 4.00 | 5.00 | x 1.00 |
| Triamcinolone | 5.00 | 4.00 | x 1.25 |

* Fludrocortisone, Aldosterone, Cloprednol, Trilostane and Cortivazol are not used for treatment of inflammatory bowel disease.
